# Proteome Profiling of Neuroblastoma-Derived Exosomes Reveal the Expression of Proteins Potentially Involved in Tumor Progression

**DOI:** 10.1371/journal.pone.0075054

**Published:** 2013-09-19

**Authors:** Danilo Marimpietri, Andrea Petretto, Lizzia Raffaghello, Annalisa Pezzolo, Cristina Gagliani, Carlo Tacchetti, Pierluigi Mauri, Giovanni Melioli, Vito Pistoia

**Affiliations:** 1 Laboratory of Oncology, Department of Translational Research and Laboratory Medicine, Istituto Giannina Gaslini, Genoa, Italy; 2 Core Facilities, Department of Translational Research and Laboratory Medicine, Istituto Giannina Gaslini, Genoa, Italy; 3 Clinical Pathology Laboratories, Department of Translational Research and Laboratory Medicine, Istituto Giannina Gaslini, Genoa, Italy; 4 Department of Experimental Medicine, University of Genoa, Genoa, Italy; 5 Experimental Imaging Center, Scientific Institute San Raffaele, Milan, Italy; 6 Institute for Biomedical Technologies, CNR, Segrate, Milan, Italy; University of Bari Medical School, Italy

## Abstract

Neuroblastoma (NB) is the most common extracranial solid tumor in childhood, with grim prognosis in a half of patients. Exosomes are nanometer-sized membrane vesicles derived from the multivesicular bodies (MVBs) of the endocytic pathway and released by normal and neoplastic cells. Tumor-derived exosomes have been shown in different model systems to carry molecules that promote cancer growth and dissemination. In this respect, we have here performed the first characterization and proteomic analysis of exosomes isolated from human NB cell lines by filtration and ultracentrifugation. Electron microscopy demonstrated that NB-derived exosomes exhibited the characteristic cup-shaped morphology. Dynamic light scattering studies showed a bell-shaped curve and a polydispersity factor consistent with those of exosomes. Zeta potential values suggested a good nanoparticle stability. We performed proteomic analysis of NB-derived exosomes by two dimension liquid chromatography separation and mass spectrometry analyses using the multidimensional protein identification technology strategy. We found that the large majority of the proteins identified in NB derived exosomes are present in Exocarta database including tetraspanins, fibronectin, heat shock proteins, MVB proteins, cytoskeleton-related proteins, prominin-1 (CD133), basigin (CD147) and B7-H3 (CD276). Expression of the CD9, CD63 and CD81 tetraspanins, fibronectin, CD133, CD147 and CD276 was validated by flow cytometry. Noteworthy, flow cytometric analysis showed that NB-derived exosomes expressed the GD2 disialoganglioside, the most specific marker of NB. In conclusion, this study shows that NB-derived exosomes express a discrete set of molecules involved in defense response, cell differentiation, cell proliferation and regulation of other important biological process. Thus, NB-derived exosomes may play an important role in the modulation of tumor microenvironment and represent potential tumor biomarkers.

## Introduction

Neuroblastoma (NB) is the most common extracranial solid tumor in childhood. It arises from primitive neuroepithelial cells of the embryonic neural crest and, therefore, tumors can develop anywhere along the sympathetic nervous system. The most frequent NB primary site is the medulla of the adrenal gland. Approximately a half of NB patients presents with metastatic disease at diagnosis involving mainly bone marrow, lymph nodes, liver and skin [1]. Despite aggressive treatment strategies, the prognosis of patients with disseminated NB is grim, with approximately 30% of them surviving at five years from diagnosis [2].

Studies on NB over the past decades have identified numerous factors that contribute to tumor progression and correlate to overall outcome including patient’s age, stage, histology, and genetic abnormalities. NB cells proliferate and colonize distant organs like the bone marrow and the bone through close interactions between tumor cells and the surrounding microenvironment [3].

The tumor microenvironment plays a key role in regulating tumor progression [4], [5], but the molecular mechanisms involved are not completely defined. In this frame, identification of proteins secreted by cancer cells is of special interest because it may allow a better understanding of tumor progression and may provide early biomarkers of disease activity.

Secreted proteins constitute an important class of molecules encoded by approximately 10% of the human genome [6]. The term “secretome” was initially used to describe the study of proteins secreted by cells, tissues or organisms that control many biological and physiological processes [7]. More recently, the definition of secretome has been broadened to include the complex of proteins released by both classical secretion mechanisms and vesicles such as exosomes [8].

The term “exosome” was used for the first time by Trams *et al*. in 1981 and originated from “exfoliated membrane vesicles” with ectoenzyme activity, secreted by normal and neoplastic cells [9].

Exosomes are nanometer-sized membrane vesicles (approximately 50–100 nm) with a cup shape morphology. Exosomes are formed by inward budding of the limiting membrane into the lumen of late endosomes (which are called multivesicular bodies or MVBs due to their multivesicular appearance) and then released into the extracellular milieu upon fusion with plasma membrane [10], [11].

Exosomes are released by both normal and neoplastic cells and are present in several biological fluids as well as in cell culture media [11], [12].

Proteomic analysis of exosomes derived from a wide variety of cells and body fluids has identified a common set of membrane and cytosolic proteins related to exosome structure and biogenesis [13]. The most conserved class of proteins associated with exosomes irrespective of the cell type from which they originate include members of the tetraspanin superfamily (e.g. CD9, CD63, CD81), heat shock proteins (Hsp70 and Hsp90), as well as proteins involved in MVBs biogenesis (alix and TSG101) and in membrane transport (annexins, flotillin, Rab GTPase) [14]. Moreover exosomes are enriched in raft-lipids, mRNA and microRNA suggesting an involvement in exchange of genetic information at local and distant sites [15], [16].

Useful databases have been developed that allow associating identified proteins to exosomes. ExoCarta (http://www.exocarta.org) is a well known database containing proteins, RNA and lipids already detected in exosomes [17].

The physiological significance of exosomes is not yet fully understood. These vesicles are involved in intercellular communication and numerous immune functions that are cell-type specific [18]. On the other hand, tumor-derived exosomes exert immunosuppressive effects such as induction of apoptosis of activated cytotoxic T cells, induction of myeloid derived suppressor cells and T regulatory cells, inhibition of the differentiation of immature to mature dendritic cells and suppression of NK cell mediated cytotoxicity. In addition, exosome-derived fibronectin triggers macrophage production of pro-inflammatory cytokines [19].

Recent studies suggest that vesicle shedding by malignant cells promotes tumor progression by stimulating neo-angiogenesis [20], carrying matrix remodelling enzymes [21], contributing to the preparation of the metastatic niche [22], and inducing antibody sequestration or drug-resistance by drug exportation [23].

In summary, these findings highlight the role exerted by tumor-derived exosomes in the generation and the modulation of tumor-supporting microenvironment [24].

In the recent years, tumor-derived exosomes isolated from several body fluids have been proposed as relevant biological sources of prognostic bio-markers obtained in a non-invasive manner [25], [26].

In this study we have performed the first characterization and proteomic analysis of exosomes derived from human NB cell lines to gain new information on the molecular mechanisms of cancer progression and to propose potential tumor biomarkers.

## Materials and Methods

### Cell Culture

The human NB cell line HTLA-230 was a gift of Dr. E. Bogenmann (Los Angeles Children’s Hospital, CA) [27]. The human neuroblastoma cell lines IMR-32 and SH-SY5Y were obtained from the American Type Culture Collection (Manassas, VA); the GI-LI-N cell line was obtained from the Biological Bank and Cell Factory (National Cancer Institute, Genoa, Italy). All cell lines were tested for mycoplasma contamination by PCR analysis [28].

All cell lines were grown as monolayers in complete medium (Dulbecco’s modified Eagle medium; Sigma), supplemented with 10% v/v heat-inactivated fetal bovine serum (Gibco-Invitrogen S.r.l., Carlsbad, CA) and 50 IU/mL of sodium penicillin G, 50 µg/mL streptomycin sulfate, and 2 mmol/L L-glutamine (all reagents from Sigma). Cells were maintained in a logarithmic phase of growth in 75 and 150 cm^2^ plastic culture flasks (BD Falcon, USA) at 37°C in a 5% CO_2_–95% air humidified incubator [29].

### Exosome Purification

The human NB cell lines HTLA-230, IMR-32, SH-SY5Y and GI-LI-N were cultured for 24 hours in complete DMEM medium supplemented with 10% exosome-free FBS (dFBS). dFBS was obtained by a pre-depletion of bovine exosomes by ultracentrifugation at 100,000 *g* for 2 hours at 4°C. Heparinized blood from healthy donors was preliminarily subjected to platelet depletion and red cells lysis, then cells were cultured for 24 hours in complete RPMI medium supplemented with 10% dFBS.

Exosomes were isolated from the supernatants of NB cell lines or of blood cells by differential centrifugation according to the method described by Théry *et al*. [30]. Briefly, culture supernatants were collected and subjected to two successive centrifugations at 300 g for 10 min and at 10,000 g for 30 min to eliminate cells and debris. The clarified medium was filtered twice with 0.22 µm and 0.1 µm pore filters, respectively (Merk Millipore, Billerica, MA), followed by ultracentrifugation at 100,000 g for 75 min (70Ti rotor, Optima L-90K Ultracentrifuge, Beckman Coulter). The resulting pellet was washed in PBS and re-ultracentrifuged twice at 100,000 g for 75 min. The final pellet was resuspended with buffer to 1/200th-1/600th of the original volume of the sample.

Purified exosomes were then quantified using the CBQCA Protein Quantitation Kit according to the manufacturer’s instructions (Molecular Probes/Invitrogen, Life Technologies Italia, Monza MB, Italy). This kit works well in the presence of lipids and can be used directly to determine the amount of proteins in lipid-protein samples. Finally, samples were aliquoted and stored at −80°C. All of the above experiments were performed three times for each cell line or blood sample.

### Dynamic Light Scattering and Zeta Potential

The potential at the surface of hydrodynamic shear of the liquid layer surrounding the particle is the so called zeta potential. Zeta potential, a physical property which is exhibited by any particle in suspension, is a measure of the magnitude of the electrostatic or charge repulsion or attraction between particles, and is one of the fundamental parameters known to affect stability.

Exosome size, polydispersity and zeta-potential were analyzed using the zetasizer nano ZS90 particle sizer at a 90° fixed angle (Malvern Instruments, Worcestershire, UK). To this end, exosomes were suspended in a solution of PBS:H_2_O (1∶10) to evaluate zeta potential and in PBS to measure size. Samples were studied at a constant temperature of 25°C. Light scattering from the sample was detected by a photomultiplier tube placed at 90° to the incident laser beam. The translational diffusion coefficient of the solutions was calculated from the time autocorrelation of the scattered light intensity and the translational diffusion coefficient was extracted from the correlogram using the method of cumulants as applied in the proprietory Malvern software. The diameter of the exosomes was obtained from the application of Stokes–Einstein equation: *d(H) = kT/3* *πηD* where *d(H)* is the hydrodynamic diameter, *k* the Boltzmann constant, *T* the temperature, *η* the shear viscosity of the solvent and *D* the diffusion coefficient of the particles [31].

### Transmission Electron Microscopy

For transmission electron microscopy (TEM) analysis, samples were prepared as described by Théry *et al*. [30]. Briefly, exosomes were fixed with 2% w/v paraformaldehyde in PBS; then they were dropped onto a formvar-carbon coated copper grids (Agar Scientific, Assing S.p.A., Monterotondo, RM, Italy) and left to dry at room temperature for 20 minutes. After a quick wash, the grids were fixed with 1% w/v glutaraldehyde in PBS, followed by several washing steps in distilled water.

Samples were contrasted by 4% w/v Uranyl Acetate and by UA-Methylcellulose mix solution.

The grid was dried at room temperature, viewed in a Tecnai 12 G2 transmission electron microscope (FEI, Eindhoven, The Netherlands) and then analyzed with an Olympus iTEM software (Olympus soft imaging solutions GMBH, Münster, Germany).

### Proteomic Analysis

#### Sample preparation

To dissolve exosome preparations, 0.5% Rapigest (Waters, Milford, MA, USA) was added to mixtures and heated at 99°C for 5 min. Mixtures were then cooled to room temperature and digested with trypsin (Sequencing Grade Modified Trypsin, Promega, Madison, WI, USA) at a 1∶50 enzyme:protein ratio (wt:wt) in 100 mM ammonium bicarbonate, pH 8.0 and incubated at 37°C overnight. Digestions were chemically stopped by acidification with trifluoroacetic acid, incubated for 40 min at 37°C, and eventually centrifuged at 13 000 g for 10 min. As a last step before analysis, samples were desalted by C18 Tips (Pierce Biotechnology, Rockford, IL, USA), and finally resuspended in 0.1% formic acid. Ten microliters of the peptide mixture were injected directly into the two-dimensional capillary chromatography-tandem mass spectrometry (2DC-MS/MS).

#### 2DC-MS/MS analysis

Ten microliters of the peptide mixtures, obtained from the digestion of the protein samples, were analyzed by means of two-dimensional microchromatography coupled with an ion trap mass spectrometer, using the ProteomeX system (ThermoElectron, San Josè, CA) equipped with Bioworks 3.1 as graphical interface for data handling. In particular, peptide mixtures were first separated by means of ion-exchange chromatography (Biobasic-SCX column, 5 µm, 0.3 ID × 150 mm, ThermoHypersil, Bellofonte, PA, USA) using 7 steps of increasing ammonium chloride concentration (0, 50, 100, 150, 200, 300, and 600 mM). Each salt step was directly loaded onto the reversed phase column (Biobasic-C18, 0.180 ID × 100 mm, ThermoHypersil, Bellofonte, PA, USA) and separated with an acetonitrile gradient: eluent A, 0.1% formic acid in water; eluent B, 0.1% formic acid in acetonitrile; the gradient profile was 5% B for 3 min followed by 5 to 50% B within 40 min. Peptides eluted from the C18 column were analyzed directly with an ion trap LTQ mass spectrometer (Thermo Fisher Scientific) equipped with a metal needle (10 µm ID). The heated capillary was held at 160°C, ion spray 3.2 kV and capillary voltage 67 V. Spectra were acquired in positive mode (in the range of 400–1600 m/z) using dynamic exclusion for MS/MS analysis (collision energy 35%).

#### Data handling of MS results

Protein identification was performed using SEQUEST software (Thermo Fisher Scientific), operating on a 10-processor computer cluster (AETHIA, Turin, Italy), and searched against a human protein database downloaded by Uniprot. Peptide MS/MS assignments were filtered following highly stringent criteria: Xcorr ≥1.9 for the singly charged ions, Xcorr ≥2.2 for doubly charged ions, Xcorr ≥3.7 for triply charged ions, peptide probability ≤0.01, delta Cn ≥0.1 and Rsp≤4.26. To identify the largest panel of peptides, the option “no enzyme” was used for the in silica digestion of human databases. Moreover, the filter for SEQUEST search was set to 1.0 for peptide tolerance. For protein assignment using data from a single peptide, we applied stringent criteria according to recently published guidelines [32]. In particular, only the first-best matching peptide was taken into consideration and only if the same peptide was found in multiple MS/MS spectra. In addition, the protein identification with a single peptide was considered valid if found in at least three of the four multidimensional protein identification technology (MudPIT) analyses. The output data obtained from SEQUEST software were treated with the Multidimensional Algorithm Protein Map in-house algorithm for comparison of the protein lists, evaluation of relative abundances, and plotting virtual 2D maps.

### Flow Cytometric Analysis

NB-derived exosomes were analyzed for the presence of several molecules by flow cytometry after vesicle adsorption onto latex beads.

In brief, 2 µg of NB-derived exosomes were incubated with 2 µL of 4 µm diameter aldehyde/sulfate latex beads (Invitrogen, Life Technologies Italia, Monza, MB, Italy) for 2 hours at room temperature; PBS supplemented with 2% FBS was then added to each sample, and the incubation was prolonged for 30 minutes.

Exosomes-coated beads were incubated for 30 min at 4°C with primary mouse anti-human FITC-conjugated monoclonal antibodies (mAbs) or with unlabeled mAbs (Merk Millipore, Billerica, MA except for anti-fibronectin, anti-B7H3 and anti-GD2 mAbs, generous gifts of Dr. L. Zardi, Prof. C. Bottino and Prof. R.A. Reisfeld respectively) plus a further incubation for 20 min at 4°C with a polyclonal rabbit anti-mouse FITC-conjugated secondary Ab (Dako Cytomation, Denmark). As negative control an isotype-matched primary mAb (Merk Millipore, Billerica, MA) was used. All antibodies were used in accordance with manufacturer instructions. Samples were then analyzed by Gallios flow cytometer and Kaluza software (Beckman Coulter, Milano, Italy).

## Results

### Purification and Physical Characterization of NB Cell-derived Exosomes

Exosomes were isolated from four NB cell line (HTLA-230, IMR-32, SH-SY5Y and GI-LI-N) culture supernatants by a combination of filtration and ultracentrifugation. These experiments were repeated three times for each cell line. The CBQCA protein quantification kit revealed that roughly 350 ng of exosomal proteins could be isolated from 1×10^6^ human NB cells after 24 h culture.

We next performed a dynamic light scattering analysis of purified exosomes in order to define their size.

**Figure 1:**
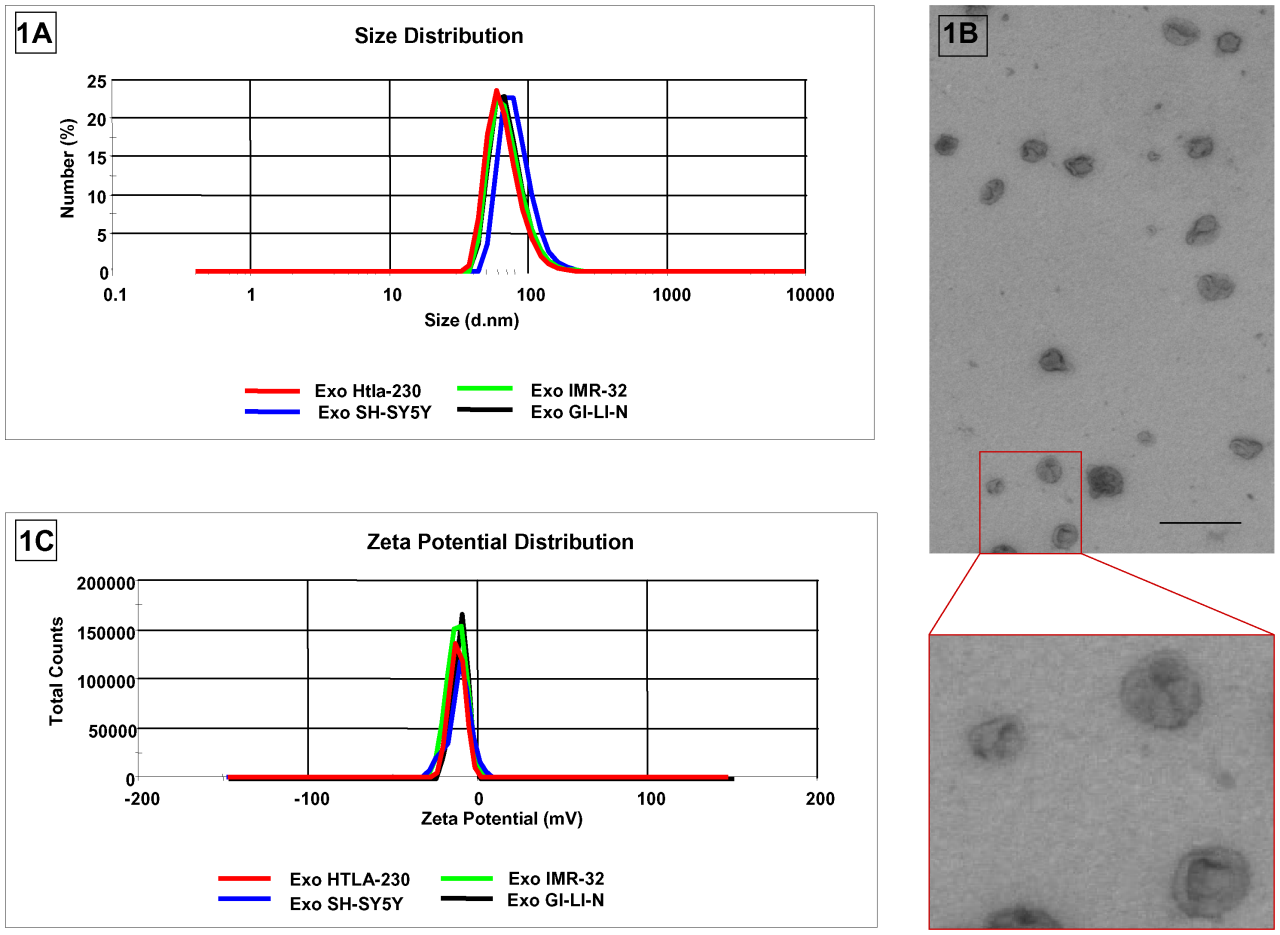
Physical charachterization of NB cell-derived exosomes. Size distribution (panel A) and Zeta Potential (panel C) of NB cell-derived nanovesicles, as assessed by the the zetasizer nano ZS90 particle sizer. Each curve shows means±SD from three replicates in a representative experiment out of the three performed with similar results. Electron microscopy analysis (panel B) of NB cell-derived nanovesicles isolated from the HTLA-230 cell line. One representative experiment out of the three performed with similar results is shown. Bar = 300 nm.

As shown in Fig. 1A, NB cell-derived exosomes formed a bell-shaped size distribution profile with a peak at 68.05±6.58 nm for HTLA-230 cells, at 72.54±8.9 nm for IMR-32 cells, at 83.66±9.22 nm for SH-SY5Y cells and at 72.20±8.83 nm for GI-LI-N cells. The size distribution profiles were similar for exosomes from the above NB cell lines, with a polydispersity factor ranging from 0.16±0.011 to 0.198±0.025, indicative of homogeneous exosome preparations (See Table 1).

**Table 1:** Physical characterization of exosomes derived from human NB cell lines.

	Size (nm)	Polydispersity	*Z*-potential (mV)
**HTLA-230**	68.05±6.58	0.167±0.007	−12.1±0.17
**IMR-32**	72.54±8.9	0.155±0.012	−14.8±1.55
**SH-SY5Y**	83.66±9.22	0.16±0.011	−13.2±1.1
**GI-LI-N**	72.20±8.83	0.198±0.025	−12.0±0.15

To further determine the size and the morphology of NB cell line-derived vesicles, we performed TEM analysis of these particles. As observed in Fig. 1B the study of HTLA-230 cell line-derived particles revealed the presence of vesicles with the typical exosome cup-shape morphology and confirmed the size obtained by dynamic light scattering analysis. Similar results were obtained with the IMR-32, SH-SY5Y and GI-LI-N cell line-derived exosome preparations (data not shown).

Moreover, to obtain information about the stability of the particles in terms of dispersion, aggregation or flocculation, we measured the zeta potential of exosomes isolated from the four NB cell lines. The zeta potential ranged from −14.8±1.55 to −12±0.15 mV (See Fig. 1 C and Table 1), suggesting a good and similar nanoparticle stability.

### Protein Identification by 2DC-MS/MS Analysis in NB Cell-derived Exosomes

HTLA-230 cells is a well known human cell line isolated from a patient with metastatic stage IV neuroblastoma [27]. These cells exhibit high levels of *MYCN* gene amplification and are used to set up a biologically and clinically relevant pseudometastatic xenograft mouse model already established in our laboratory [29]. The exosomes derived from HTLA-230 cell line fully represent the exosomes derived from the other NB cell lines in terms of physical characterization, as shown in Figure 1 and Table 1.

**Figure 2:**
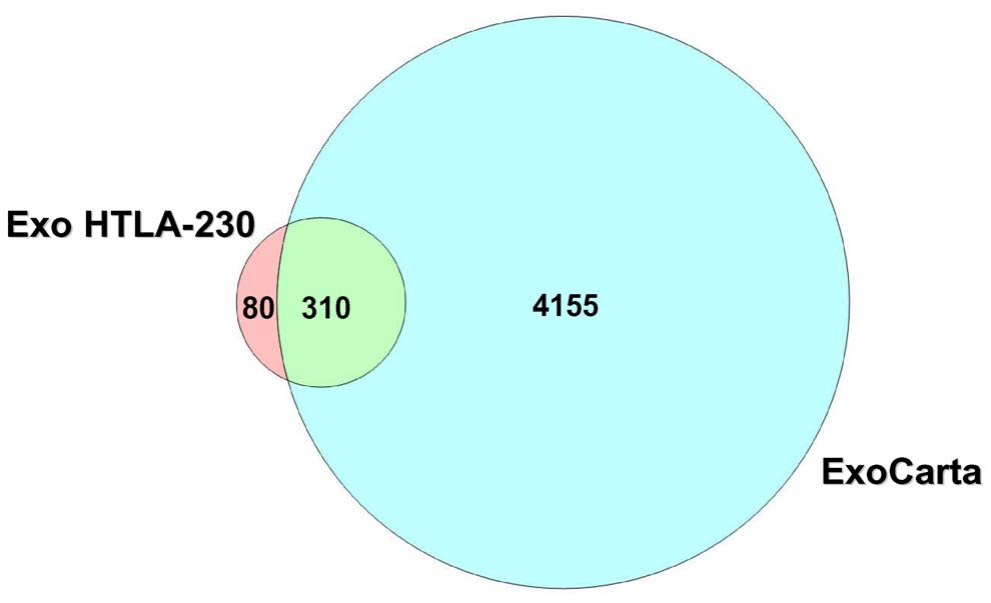
Venn diagram. Venn diagram showing protein profile overlap between HTLA-230 derived exosomes and the ExoCarta database, based upon the analytical data shown in Table S1. Nearly 80% of the proteins identified in HTLA-230 derived exosomes are present on ExoCarta database.

Exosomal proteins derived from HTLA-230 NB cell line were analyzed by 2DC-MS/MS, and 390 proteins were identified using SEQUEST software. The two highest score values were found for the pro-inflammatory protein Fibronectin and the major protein of the polyhedral coat of coated pits and vesicles Clathrin Heavy Chain, with scores of 410.31 and 404.34 respectively. Both proteins are reported in the ExoCarta database as exosome-associated [19], [33]–[35]. Altogether, 310 out of 390 (79.5%) proteins identified in NB derived nanoparticles were present on the latter database (Table S1), as shown in the Venn diagram in Fig. 2, indicating that the vesicles isolated from the HTLA-230 cell line had a protein composition consistent with that of exosomes.

A proportion of the 80 proteins detected in NB-derived exosomes, but not in the ExoCarta database (Table S1), may represent a “signature” of cells of neuroblastic origin. Other unmatched proteins, such as a few tubulin family members identified in HTLA-230 cell-derived exosomes only (e.g. tubulin 1 beta), have close homologues that are listed in the ExoCarta database (e.g. tubulin 3 beta).

**Figure 3:**
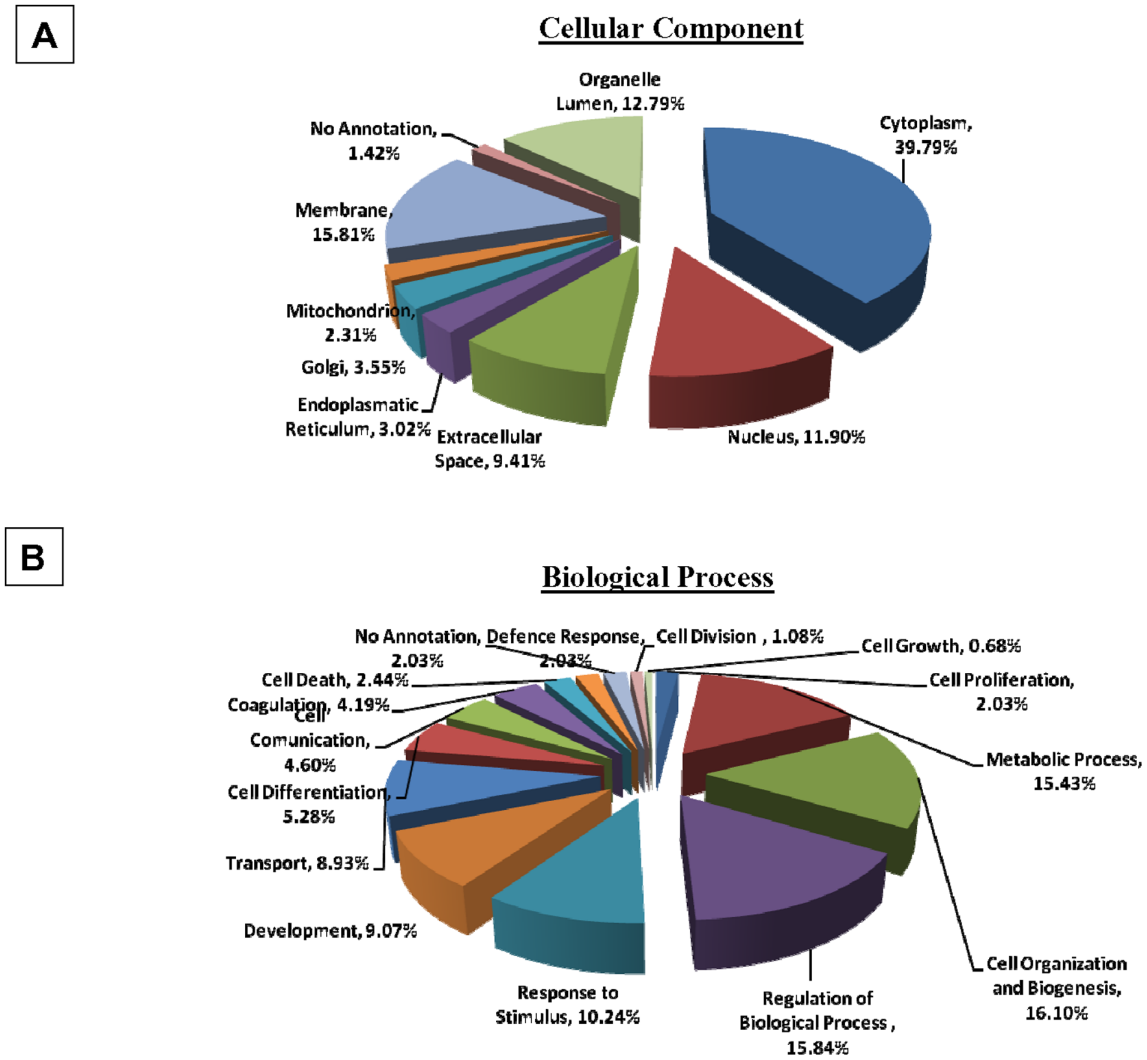
Categorizations of NB-derived exosomal protein. Categorizations of protein identified in HTLA-230 derived exosomes by cellular component (Figure 3A) and biological process (Figure 3B) based upon the analytical data shown in Table S1.

Exosome-derived proteins were then categorized by cellular component (Fig. 3A) and biological process (Figure 3B) as percentages of the total number of identified proteins using UNIPROT database and Perseus software for statistical analysis.

Proteins associated by cellular components showed the highest percentage of cytoplasmic-derived proteins (39.8%), with relatively lower percentages of membrane proteins (15.8%). Intriguingly, these distributions indicated high proportions of organelle lumen- and nucleus-derived proteins, respectively with 12.7% and 11.9% (Fig. 3A). However, it must be noted that several molecules such as Prominin 1 (CD133) or some Heat Shock Proteins may have multiple subcellular localizations [36], [37].

Classification of exosomal proteins by biological process showed a large number of categories, suggesting that NB-derived exosomes could be implicated in different functions. Nevertheless, the system is redundant since several proteins may be involved in multiple biological processes. The largest category is represented by proteins involved in metabolic processes (15.5%), followed by proteins involved in cell differentiation (5.3%), cell proliferation (4%), cell death (2.5%) or defense response (2%) (Fig. 3B).

### Validation of Selected Proteins in NB Cell-derived Exosomes by Flow Cytometry

In order to validate selected proteins detected in the proteomic profile, we performed a flow cytometric analysis of NB-derived exosomes bound to aldehyde/sulphate latex beads.

**Figure 4:**
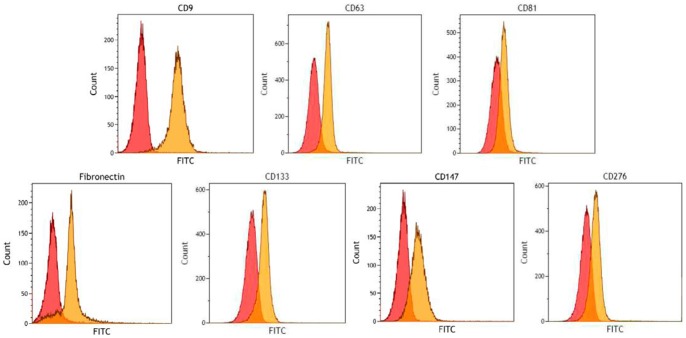
Validation of selected protein expression by flow cytometry. Flow cytometry analysis of selected protein expression in exosomes purified from the supernatant of HTLA-230 cells and coated to latex beads. CD9, CD63 and CD81 tetraspanins as well as fibronectin, prominin-1/CD133, basigin/CD147 and B7-H3/CD276 were tested. One representative experiment out of the three performed with similar results is shown.

Fig. 4 shows the results of a representative experiment out of the three performed with similar results. The latex bead cytofluorimetric assay revealed good levels of signals for the three tetraspanin molecules (CD9, CD63 and CD81) tested as exosomal markers on vesicles derived from HTLA-230 NB cell line. Furthermore, the presence of the pro-inflammatory protein Fibronectin, the putative cancer stem cell marker Prominin-1 (CD133), the tumor cell-derived collagenase stimulatory factor Basigin (CD147) and the immunosuppressive molecule B7-H3 (CD276) was confirmed by flow cytometric analysis of exosome-coated beads (Fig. 4). The same proteins were detected by flow cytometry on the HTLA-230 NB cell line (not shown).

### GD2 Detection in NB Cell-derived Exosomes

The GD2 ganglioside (disialoganglioside) is considered the most specific NB marker. It is expressed on the surface of human NB cells as well as in plasma of NB patients [38]. Exocarta database and several recent studies indicate that exosomal membrane contains different lipid molecules including gangliosides [33], [39].

**Figure 5:**
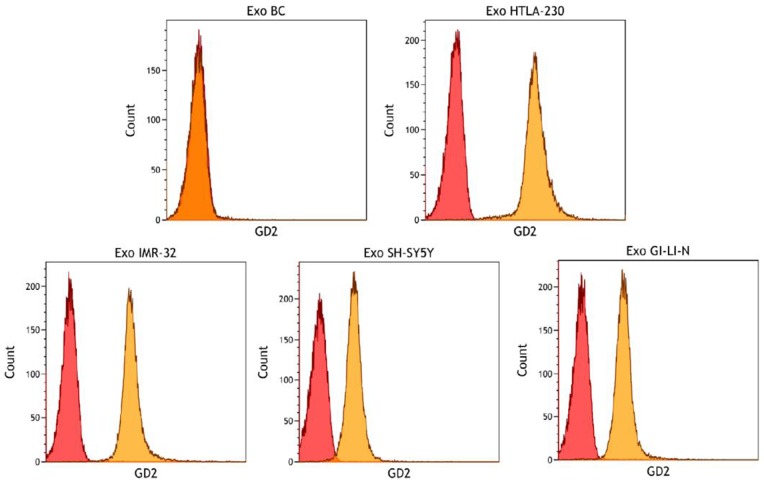
Detection of GD2 molecule in NB cell-derived exosomes. Flow cytometry detection of GD2 molecule on exosomes derived from Buffy Coat (BC) and NB cell lines adsorbed onto latex beads. One representative experiment out of the three performed with each cell line with similar results is shown.

To test the hypothesis that GD2 ganglioside was contained in NB-derived exosomal membrane, we performed a specific cytofluorimetric assay on tumor exosomes derived from a panel of human NB cell lines (HTLA-230, IMR-32, SH-SY5Y and GI-LI-N) and exosomes isolated from healthy donor blood cell populations, both pre-adsorbed onto latex beads. As expected, strong expression of the GD2 molecule was detected in exosomes derived from all NB cell lines but not from normal blood cells (Fig. 5). Accordingly, HTLA-230, IMR-32, SH-SY5Y and GI-LI-N cell lines, but not blood cells, expressed GD2 on the cell surface, as assessed by flow cytometry (not shown).

## Discussion

Cell interactions play a pivotal role in shaping the tumor microenvironment. Transformed cells exchange signals with surrounding fibroblasts, endothelial cells and immune cells through both direct cell-to-cell interactions and secreted molecules. Thus, tumors can release growth factors for endothelial cells, or chemokines and cytokines dampening anti-tumor immune responses [40]. Active release of vesicles is another mechanism whereby tumor cells interact with stromal cells leading to activation of surface receptors and delivery of molecules stored in the vesicles into adjacent cells [16].

Exosomes represent a distinct class of nanometer-sized membrane vesicles of endocytic origin which expose transmembrane receptors and contain proteins and RNA derived from the secreting cells. Exosomes are released in different biological fluids such as plasma, urine, amniotic fluid, breast milk, bronchoalveolar lavage and synovial fluids, under both physiological and pathological conditions [13].

Several studies have been carried out on exosomes from different malignancies such as melanoma [41], prostate cancer [42], bladder cancer [34], colorectal cancer [43] and others. Due to the abundant expression of tumor-associated antigens, tumor-derived exosomes have been considered as source of antigenic determinants to be exploited in the design of cancer vaccine strategies [44], [45].

To the best of our knowledge, this is the first report on the characterization of nanovesicles released by four human NB cell lines, i.e. HTLA-230, IMR-32, SH-SY5Y and GI-LI-N. The exosomal nature of these nanovesicles was unambiguously proven by size, shape and presence of canonical proteins. These exosomal preparations were stable, as shown by the analysis of zeta potential in physiologic pH.

Proteomic characterization of exosomes derived from the HTLA-230 cell line allowed the identification of 390 proteins. Approximately 80% of such proteins are present in Exocarta database, derive mainly from cytoplasm and are involved in various metabolic processes. Interestingly, a portion of the 20% of NB-derived exosomal proteins not present in the Exocarta database, such as MDK, DBH, CNTN1, NCAM1, CNTFR, PLXNA3 and ADRB2, may represent a “signature” of cells of neuroblastic origin (see Table S1).

The highest scores were attained by Fibronectin, a consistent component of exosomes derived from both normal and tumor cells [33], [34], and Clathrin, the major protein of the polyhedral coat of coated pits and vesicles [35]. Exosomal fibronectin was previously shown to induce IL-1β production by macrophages [19], thus contributing to create an inflammatory microenvironment that supports tumor growth. In this respect, NB infiltration with inflammatory cells has been recently correlated to unfavourable prognosis [46].

Clathrin-coated vesicles are formed at the plasma membrane, where they select protein and lipid cargo for endocytic entry into cells. These vesicles are also formed at the trans-Golgi network, where they function in protein transport from the secretory pathway to the endosomal/lysosomal system [47]. These findings could explain not only the pivotal role of clathrin in the formation of MVBs, but also the presence of several surface membrane molecules in the protein profile of exosomes.

Flow cytometry analysis of HTLA 230-derived exosome-coated beads confirmed the expression of a subset of cell surface molecules involved in tumor-supporting microenvironment. Thus, Prominin-1 (CD133) is a pentaspan membrane protein containing five transmembrane domains, two large N-glycosylated extracellular loops, two small intracellular domains and a cytoplasmic C-terminal domain [48]. CD133 was reported to be located into the membrane microdomains forming active transduction complexes [49] and could play a role in cell polarity and integration *via* cell-to-cell and cell-matrix interactions [50]. Moreover, although its function remains unclear, Prominin-1 seems to be involved in maintaining stem cell properties, such as symmetric/asymmetric balance division [51]. Noteworthy, CD133 is widely used as a cancer stem cell marker and its expression may be associated with poor outcome in NB [52]. Accordingly, Prominin-1 was shown to suppress NB cell differentiation via signal pathway modification [53]. Prominin-1 may be released from neural progenitors by budding of plasma membrane protrusions and/or transported in exosomes [36].

B7-H3 (CD276) is a type I transmembrane protein sharing 20%-27% amino acid homology with other B7 family members [54]. Both stimulatory and inhibitory B7-H3 functions have been identified, and its expression in cancer cells has been related to contrasting effects on tumor growth [55]. CD276 is a NB-associated molecule that protects cancer cells from the attack of natural-killer cells [56]. It has been proposed that B7H3 may be used as NB-specific marker in the bone marrow and help in the identification of NB patients at risk of relapse [57].

Recent studies have also reported that B7-H3 is released from early and term placenta via exosomes, with important implications in the mechanisms whereby trophoblast immunomodulators modify the maternal immunological environment [58].

Basigin (CD147 or EMMPRIN) is a multifunctional transmembrane glycoprotein with two immunoglobulin-like domains involved in lymphocyte responsiveness, reproduction, neural function and inflammation. EMMPRIN is also known to be an upstream inducer of several extracellular metalloproteinases and is suggested to be the master regulator of metalloproteinases production in pathological processes such as tumor invasion and metastasis [59], [60].

Interestingly, Basigin has been identified as a marker of invasion and/or poor prognosis in numerous solid tumors, such as melanoma, prostate, breast, head and neck squamous cell carcinoma, and ovarian cancer [61]–[66]. Inhibition of Basigin expression in both pancreatic cancer cells and a glioblastoma cell line reduced tumor cell invasion, angiogenesis, metastasis and increased chemosensitivity [67], [68]. Thus, our findings prompt additional studies to investigate the role of basigin in NB growth and invasiveness, as well as the potential relevance of CD147 as a new biomarker in NB patients.

Tumor gangliosides are membrane glycosphingolipids with immunosuppressive activities shed into the tumor microenvironment [69]. GD2 ganglioside, a derivative of cerebroside containing two sialic acid residues, is expressed on most NB tumors and is involved in the inhibition of T, NK and dendritic cell function [70], [71]. The presence of the GD2 molecule was further demonstrated in plasma of NB patients [38]. Recently, gangliosides have been detected on exosomal membrane in several studies [33] and this has been confirmed in ExoCarta database. Yu Kong *et al*. [72] reported that the pellet of membrane vesicles isolated from a murine lymphoma cell line contained nearly one-third of total shed gangliosides. In this vein, we demonstrate here that exosomes derived from a panel of human NB cell lines highly express the GD2 ganglioside molecule.

In conclusion, we have carried out the first proteomic characterization of NB cell-derived exosomes and identified a number of proteins that modulate the tumor microenvironment and promote tumor progression. The finding that these exosomes express the NB-specific ganglioside GD2 combined with the notion that circulating tumor-derived exosomes increase with tumor progression [73] suggest that blood GD2^+^ exosomes may represent a novel potential biomarker for human NB.

## Supporting Information

Table S1 **List of all proteins identified in NB-cell derived exosomes.** Underscored proteins represent those present in Exocarta database. Data shown refer to a representative experiment out of the four performed with similar results.
(XLS)
